# Choice of healthcare provider following reform in Vietnam

**DOI:** 10.1186/1472-6963-8-162

**Published:** 2008-07-30

**Authors:** Nguyen Thi Bich Thuan, Curt Lofgren, Lars Lindholm, Nguyen Thi Kim Chuc

**Affiliations:** 1 Planning and Financing Department of Ministry of Health, Hanoi, Vietnam; 2 Umeå International School of Public Health, Umeå University, Umeå, Sweden; 3 Public Health Faculty, Hanoi Medical University, Hanoi, Vietnam

## Abstract

**Background:**

In Vietnam, the health-sector reforms since 1989 have lead to a rapid increase in out-of-pocket expenses. This paper examines the choice of medical provider and household healthcare expenditure for different providers in a rural district of Vietnam following healthcare reform.

**Methods:**

The study consisted of twelve monthly follow-up interviews of 621 randomly selected households. The households are part of the FilaBavi project sample – Health System Research Project. The heads of household were interviewed at monthly intervals from July 2001 to June 2002.

**Results:**

The use of private health providers and self-treatment are quite common for both episodes (60% and 23% of all illness episodes) and expenditure (60% and 12.8% of healthcare expenditure) The poor tend to use self-treatment more frequently than wealthier members of the community (31% vs. 14.5% of illness episodes respectively). All patients in this study often use private services before public ones. The poor use less public care and less care at higher levels than the rich do (8% vs.13% of total illness episodes, which decomposes into 3% vs. 7% at district level, and 1% vs. 3% at the provincial or central level, respectively). The education of the patients significantly affects healthcare decisions. Those with higher education tend to choose healthcare providers rather than self-treatment. Women tend to use drugs or healthcare services more often than men do. Patients in two highest quintiles use health services more than in the lowest quintile. Moreover, seriously ill patients frequently use more drugs, healthcare services, public care than those with less severe illness.

**Conclusion:**

The results are useful for policy makers and healthcare professionals to (i) formulate healthcare policies-of foremost importance are methods used to reduce self-treatment and no treatment; (ii) the management of private practices and maintaining public healthcare providers at all levels, particularly at the basic levels (district, commune) where the poor more easily can access healthcare services, is also important, as is the management of private practices and (iii) provide a background for further studies on both short and long-term health service strategies.

## Background

Access to healthcare providers is a significant factor in improving public health and helping poor households escape from poverty [1]. In developing countries, out-of-pocket payments for health services have catastrophic economic effects on individuals and their ability to seek and receive adequate healthcare [2,3]. The choice of healthcare service depends on the various characteristics of potential providers (e.g., area of expertise or quality of service), as well as of the patients themselves (e.g., economic status, health status, education, age, and gender). Such factors can influence accessibility to healthcare, even where services exist [4].

This paper discusses (a) the current pattern of healthcare in a rural district of Vietnam in relation to the choice of provider, and (b) household expenditure for different providers following the transition from a socialist system to a market economy in Vietnam from 1989 until 2002. During this period, the Vietnamese economy grew rapidly and the living standards of both urban and rural areas improved significantly.

Coupled with this growth was a rapid widening of the economic gap between the rich and poor [5]. Vietnam is still a poor country. While the annual income per capita has increased by 5–6%, the average annual income remained around USD370 in 2002 [6,7]. The national budget allocated for healthcare is still limited to approximately USD5.7 (VND91,100) per capita per year in 2002 [8]. Therefore, the government has implemented a number of measures to mobilize new resources for the heath sector. Among the most important measures have been the introduction of user fees at public hospitals, health insurance schemes, the legalization of the pharmaceutical industry, and the deregulation of the retail trade in drugs [5,9,10].

Under these measures, the government has given considerable autonomy to healthcare providers, and has relaxed commercial sales of pharmaceuticals and fee-for-health service. These changes have led to significant improvements in the quality of Vietnam's healthcare [11,12] such as the use of different providers and increased spending for healthcare. However, they have also lead to an increase the out-of-pocket health expenditures as a proportion of total health expenditures from 59% in 1989 to 84% 1998 and 80% in 2001 [10,13] which has resulted in a portion of the population being unable to afford utilize healthcare services [14].

Some surveys show that self-treatment and the use private of providers are very common among the rural households. The poor report "no treatment" or the use of commune health centers more often than the non-poor. They used less public care and less care at higher levels than the rich do, and paid as much as the better-off did when visiting public healthcare facilities [22,24].

The purpose of this paper is to assess the affect of these various policy changes on (i) the pattern of medical provider choice for rural Vietnamese residents, and (ii) the factors that affect the choice of healthcare provider and the allocation of household expenditure to different (types of) healthcare provider for different expenditure groups.

These findings should be of interest to policy-makers and health professionals in formulating and implementing intervention policies.

## Methods

This study was conducted in the Bavi district of Hatay province in Vietnam. Bavi district is situated in the Northwest part of Vietnam, which about 60 km west of Hanoi. It has a population of 235,000 comprising three major ethnic groups: Kinh (91%), Muong (8%) and Dao (1%). There are also some families of the Tay, Hoa, and Khmer tribal groups. The district is divided into 32 communes including one small town. Farming is the predominant occupation [13,14].

There is a demographic surveillance site in Bavi, the Epidemiological Field Laboratory for the Health Systems Research Project (Filabavi) in Vietnam. FilaBavi is a joint project between Hanoi Medical University, Karolinska Institute, University of Umeå, and the Nordic School of Public Health in Sweden. The goals of the project are to implement a longitudinal epidemiological surveillance system to collect basic health and health care data, to supply information for health planning, to serve as a background and a sampling frame for specific studies (especially intervention studies), and to constitute a setting for epidemiological training of research students. In 1999 a baseline household survey was conducted followed by quarterly surveillance of vital events and complete re-surveys every two years [14].

The FilaBavi infrastructure was utilised for the study presented in this paper. The total sample consists of 11,089 households which were selected using a multistage sampling procedure. In the first stage, 67 population clusters were selected using a probability proportional to size. These clusters had 11,089 households and 51,024 individuals [14]. Assuming an α level of 5% and a 50% probability that a household will have an episode of illness in a year, the give a sample size of 576. To ensure adequate sample size, one out of every 18 households was randomly selected from the original sample. The procedure generated a sample of 629 households.

The study units of the FilaBavi project are households. The heads of households were interviewed at monthly intervals during July 2001 to June 2002. If the head of the household could not be contacted, another adult was interviewed. These household representatives provided information on each member of household's health situation, healthcare utilisation, household health expenditures as well as total incomes and expenditures each month for 12 months. For information on illnesses, the respondents were specifically asked if the household member in question had seen a medically trained person (doctor, nurse, health worker or such) and if so, what diagnosis had been made. For all questions related to female-specific diagnoses, the interviewers were instructed to interview the patient directly.

Households kept daily notes of their health situation and healthcare payments. Such notes included illness events for every person in the household as well as household incomes and expenditures. During the first week of each month, the interviewer conducted an interview based on the daily notes from the previous month. The interviews were carried out by 42 qualified interviewers employed by the larger FilaBavi project. All interviewers had completed high school and were inhabitants of the Bavi district. The interviewers used a structured questionnaire and were given special training on data collection strategies for collecting information on income, expenditure and illnesses. A systematic random sampling approach was applied in the study; about five to ten per cent of the questionnaires were randomly selected for re-interviews and for checks by researchers before the data entry.

Microsoft ACCESS™ was used for data entry. Data analyses were done with STATA (Stata Corporation, College Station, TX, USA) software.

In the analysis, statistical significance was deemed to be at a 95% confidence level (CI) or p-value < 0.05. A multiple logistic regression method was used to identify the factors influencing the use of drugs or services. The choice of healthcare provider was based on the use of healthcare for multiple episodes of illness on the same individuals. Hence, when analyzing the data using multiple logistic regression method we have adjusted standard errors for correlation between episodes for each person (corrections for clustering).

The expenditure quintile (hereinafter referred to as quintile) is ranked by equalized per capita household expenditure (eqexp_h_). The following analysis utilizes expenditure groups when comparing indicators by income category. To identify the quintile we use the methodology developed by Xu. (2005): eqexp_h _= exp_h_/eqsize_h_;

Where eqsize_h _= hhsize_*h*_^*β*^

*exp*_*h *_is household expenditures; *eqsize*_*h *_is the equivalence scale, *hhsize*_*h *_is the average of household size or number of household members.

The value of the parameter β (0.56) has been estimated from previous studies based on household survey data from 59 countries [19]. The equivalent household size for each household is generated by the formula: eqsize_h _= hhsize_*h*_^*0.56*^.

### Definitions used in this study

To be considered in this study, an *illness episode *must meet at least one of the following criteria: the subject stayed in bed; had been restricted from normal activities (e.g. work or school); or had been able to do normal activities but with reduced capacity for at least one day. An illness episode concludes when normal activities resume [17].

*Symptoms *are defined as a perceptible change in the body. The study considered the four most prevalent symptoms to include cough, fever, headache, and "pain in bones or joints". During an illness episode, a person might have more than one symptom.

*Diseases *are reported based upon the diagnosis notes collected by the health workers.

*Illness *is the occurrence of at lease one of the above symptoms or diseases during an illness episode.

*Perceived seriousness of illness *is classified into three levels: *"can work,"* where the patient was ill but could still be at work; *"miss work,"* where they are absent from work but can move around during the illness;*"confined to bed,"* where they are bedridden, an invalid, or incapacitated to such an extent that they must depend on a care giver.

*Medical provider *is classified as a traditional healer, a community health station (CHS), district health centre (DHC), province/centre hospital (P/CH), or private healthcare facility.

*Self-treatment *is an action whereby patients treat themselves using medicines available at home, purchased from drug sellers without any medical examination, or taken following the advice given by any person without formal medical background [17].

*Traditional healer treatment *is treatment whereby patients receive healthcare or take traditional medicines from traditional healers.

*Private healthcare *includes healthcare services provided by private clinics, by public health workers when they are practicing privately after regular work or are retired health workers practicing at home, or services/medication purchased following medical examination or advice given by drug sellers.

*Educational level *is classified into three categories: (i) *no-schooling *– never attended school; (ii) *only primary school *– less than 7 years (with the pre-reform education system) or 9 years (with the post-reform system) of education; (iii) *higher education *– high school graduation, university study, graduation, or vocational education after high school.

*Household expenditure *is measured by cash payments for different purposes (including the interest due on borrowed funds but not including payment on the original debt).

*Socio-economic status (SES) *is classified by household total expenditure quintiles. Expenditure, rather than income, is commonly used as a measure of SES in developing countries for three reasons. First, expenditure more accurately reflects the basic purchasing power of the household. Second, households may be less willing to state their true income or may underestimate their total income. Third, expenditure may vary less over time than income, therefore it is easier to measure [15,18].

The division into expenditure quintiles is based on data from the sum of twelve months' expenditures. The expenditures recorded in the study are the total financial outlays that the households had each month for food, health care and other means.

### Ethical considerations

This study was approved by the Scientific and Ethical Committee, Hanoi Medical University, and the Ministry of Health (Decision -QD-BYT-2001). The study was also agreed to by the local authorities, and heads of households.

## Results

Based on the survey data, there were 8,380 illness episodes reported by 2,727 individuals in 621 households. Average household size is 4.4. Health services or drugs were used to treat almost all of the illness episodes (97%).

The data shows that private providers were the most common form of curative service used by study participants (almost 60% of episodes). Public providers were used in only 10% of the episodes. Services for the remaining illness episodes consisted of self-treatment (approximately 23%) and a few mixed services or use of drugs (less than 5%). Self treatment was more common among the poor (31%) than among the rich (14.5%). For public providers, people in the highest quintile used DHC, P/CH more often than those in the lowest quintile. Similarly, for the perceived seriousness of an illness, the rich more regularly reported their illness to be less critical (*can work *and *miss work*) than the poor. A more serious illness (*confined to bed*) was reported significantly more often (40% vs. 24%) by those in the lowest quintile than by those in the highest quintile (Table 1).

**Table 1:** The use of providers for the episodes of illness (%)

	Equalized household expenditure quintiles					Total	p-value
			
	Poorest	2	3	4	Richest		
No drug or service used	76(5.0)	53(3.3)	53(2.9)	56(3.1)	17(1.0)	255(3.0)	**
Self treatment	470(31.0)	435(27.2)	480(26.7)	283(15.7)	242(14.5)	1910(22.8)	**
Private providers	776(51.1)	887(55.5)	1060(58.9)	1170(65.1)	1105(66.4)	4998(59.6)	
Traditional healer	63(4.2)	54(3.4)	107(5.9)	172(9.6)	190(11.4)	586(7.0)	**
Privatehealth care	713(47.0)	833(52.1)	953(52.9)	998(55.5)	915(55.0)	4412(52.6)	
Public providers	129(8.5)	128(8.0)	136(7.6)	220(12.2)	225(13.5)	838(10.0)	
Commune health station	62(4.1)	56(3.5)	71(3.9)	66(3.7)	51(3.1)	306(3.7)	**
District health centre	47(3.1)	55(3.4)	35(1.9)	98(5.5)	119(7.2)	354(4.2)	**
Province/central hospital	20(1.3)	17(1.1)	30(1.7)	56(3.1)	55(3.3)	178(2.1)	**
Mixed all	67(4.4)	96(6.0)	72(4.0)	69(3.8)	75(4.5)	379(4.5)	
Total number of episodes	1518(100)	1599(100)	1801(100)	1798(100)	1664(100)	8380(100)	
Perceived seriousness of illness							
Can work	495(32.6)	569(35.6)	662(36.8)	702(39.0)	652(39.2)	3080(36.8)	
Missed work	420(27.7)	532(33.3)	635(35.3)	654(36.4)	613(36.8)	2854(34.1)	**
Confine to bed	603(39.7)	498(31.1)	504(28.0)	442(24.6)	399(24.0)	2446(29.2)	***
Total number of episodes	1518(100)	1599(100)	1801(100)	1798(100)	1664(100)	8380(100)	
Total number of persons	479(100)	558(100)	577(100)	558(100)	555(100)	2727(100)	

HH is the abbreviation of household; p-value for the comparison between the lowest and highest quintile groups; *** denotes significant at the 1% level; **denotes significant at 5% level. Percentage shares are shown within brackets.

For episodes of illness in which individuals used healthcare services, the rich used services more frequently than the poor (approximately 84%vs.66%). More significantly, self-treatment accounted for 32.6% of the cases in the poorest group, compared to 14.7% in the highest quintile group (see Table 2).

**Table 2:** Episodes of illness for which a provider was used according to the choice of provider (%)

	Equalized household expenditure quintiles					Total	p-value
			
	Bottom	2	3	4	Top		
Used drugs or services							
Used service (public/private)	956(66.3)	1,088(70.4)	1,254(71.7)	1,440(82.7)	1,385(84.1)	6,123(75.4)	**
Self-treatment	470(32.6)	435(28.1)	480(27.5)	283(16.2)	242(14.7)	1,910(23.5)	**
M ixed all	16(1.1)	23(1.5)	14(0.8)	19(1.1)	20(1.2)	92(1.1)	
Total	1,442(100)	1,546(100)	1,748(100)	1,742(100)	1,647(100)	8,125(100)	

HH is the abbreviation of household; p-value for the comparison  between the lowest and highest quintile groups; *** denotes significant at the 1% level; **denotes  significant at 5% level. Percentage shares are shown within brackets.

The influence of various socio-demographic variables and perceived seriousness of illness on the decision to use drugs/services and the decision to choose a medical provider versus self-treatment and a private versus a public provider when ill was analysed using multivariate logistic regression. The finding is provided in Table 3.

**Table 3:** Multivariate logistic regression showing variables influencing the odds of using services or drugs and the choice of providers when being ill

	Using vs. not use services or drugs	Providers vs. Self-treatment	Public vs. private
Explanatory variables	OR (CI 95%)	OR (CI 95%)	OR (CI 95%)

	(N = 8380)	(N = 8125)	(N = 6123)
	R2: 0.085	R2: 0.036	R2:0.049

No of household members (continuous)	1.07(0.98–1.16)	**1.05(0.01–1.11)**	0.98(0.88–1.07)
Perceived seriousness of illness			
Can work (reference category)	1.00	1.00	1.00
Miss work	**2.70(1.95–3.73)**	**1.31(1.15–1.5)**	1.31(0.89–1.91)
Confine to bed	**4.55(3.0–6.98)**	**1.85(1.59–2.16)**	**3.11(2.23–4.35)**
Male (reference group female)	**0.49(0.34–0.71)**	0.96(0.81–1.13)	0.87(0.72–1.04)
Age group			
15–60 (reference category)	1.00	1.00	1.00
Under 15	1.59(94–2.67)	1.13(0.89–1.43)	1.41(0.94–2.09)
Over 60	0.71(0.45–1.10)	1.17(0.94–1.58)	**0.41(0.25–0.78)**
Education level			
No schooling (reference category)	1.00	1.00	1.00
Only primary school	**2.10(1.29–3.42)**	1.22(0.94–1.58)	0.80(0.50–1.28)
Higher education	**3.59(1.65–6.79)**	**1.51(1.05–2.16)**	1.12(0.61–2.05)
Equalized household expenditure quintiles			
Poorest (reference category)	1.00	1.00	1.00
2nd	1.55(0.96–2.46)	1.22(0.95–1.56)	1.20(0.68–2.08)
3rd	**1.67(1.02–2.72)**	**1.31(1.02–1.66)**	1.34(0.79–2.23)
4th	**1.66(1.11–2.83)**	**2.51(1.92–3.28)**	**1.89(1.11–3.23)**
Richest	**4.78(2.42–8.43)**	**2.77(2.10–3.66)**	**2.09(1.22–3.32)**

Adjusted standard errors for correlation between episodes for each person (corrections for clustering). See earlier the method section; bold represents significance.

The number of people using health services or drugs increased from the lower to higher education level (2.10, CI: 1.29–3.42 and 3.59, CI: 1.65–6.79, respectively). Those who perceived their illness to be sufficiently serious to *miss work *(2.7, CI: 1.95–3.73), or those in the *confined to bed *category (4.54, CI: 3.00–6.98) were also more likely to use health services or drugs than those who could still work during their illness episode. Individuals in the highest quintile were almost 5 times more likely to use health services or drugs than those in the lowest quintile.

Patients who had a higher education were one and half times more likely to use a provider versus self-treatment than those with no schooling (1.51, CI: 1.05–2.16). The number of people making such decisions increased from not at all serious to very serious scales (1.31, CI: 1.15–1.5 and 1.85, CI: 1.59–2.16), and from the third to the highest quintiles groups. Furthermore, when the household size increased by one person, the odds of choosing a provider increased by 5%. (1.05, CI: 1.01–1.11)

The choice of public versus private provider correlated significantly with age in group 60+ and being confined to bed by the perceived seriousness of illness. Individuals in two highest quintiles were almost two times more likely to use such health services than those in the lowest quintile.

In addition, the distribution of household curative expenditures for self-treatment and for different providers is presented in Table 4. The results show that the highest percentage of healthcare payments was for private services (59.9%). The share of payments was for self-treatment (12.8%); these payments were significantly higher in the lowest quintile (17.3%) group when compared with the highest quintile (8.7%) group. The percentage of payments for treatment in DHC and P/CH were significantly higher in the highest quintile than in the lowest quintile groups (7.6% vs. 6.6% and 22.4% vs. 9.8%, respectively). By contrast, the share of payments for treatment in community health station was significantly lower in the highest quintile than in the lowest quintile group (2.2% vs. 6.3% (Table 4).

**Table 4:** Household curative expenditures per year by providers and expenditure quintiles in Vietnamese dong (%)

	Equalized household expenditure quintiles					Total	p-value
			
	Bottom	2	3	4	Top		
Self-treatment	44,930(17.3)	54,707(14.5)	71,020(14.1)	68,341(13.6)	62,575(8.7)	60,332(12.8)	**
Provider(Public+private)	714,930(82.7)	322,547(85.5)	432,668(35.9)	434,280(86,4)	658,559(91.3)	412,615(87.2)	**
Private providers	155,922(60.0)	214,627(56.9)	292,527(58.1)	326,895(65.0)	426,687(59.2)	283,346(59.9)	
Traditional healer	19,962(7.7)	21,626(5.7)	45,266(9.0)	53,294(10.6)	40,181(5.6)	36,081(7.6)	**
Private healthcare	135,960(52.3)	193,001(51.2)	247,261(49.1)	273,602(54.4)	386,506(53.6)	247,266(52.3)	
Public providers	58,938(22.7)	107,920(28.6)	140,141(27.8)	107,385(21.4)	231,872(32.2)	129,269(27.3)	**
Commune health station	16,360(6.3)	19,848(5.3)	46,813(9.3)	19,328(3.8)	15,961(2.2)	23,699(5.0)	**
District health centre	17,034(6.6)	65,697(17.4)	52,600(10.4)	38,198(7.6)	54,522(7.6)	45,621(9.6)	**
Province/central hosp ital	25,544(9.8)	22,375(5.9)	40,728(8.1)	49,859(9.9)	161,389(22.4)	59,948(12.7)	***
Total curative	259,790(100)	377,255(100)	503,689(100)	502,622(100)	721,134(100)	472,947(100)	

HH is the abbreviation of household; p-value for the comparison  between the lowest and highest quintile groups; *** denotes significant at the 1% level; **denotes  significant at 5% level. Percentage shares are shown within brackets.

## Discussion and Conclusion

Monthly data collection and interview-based follow-ups tracked households for the period of one year. We believe this to be a particular strength of the study because we have avoided the inherent bias in 4-week cross-sectional surveys that can be affected by seasonal fluctuations. The head of household, using daily notes (a system used to assure accuracy in reporting), reported illness events and household healthcare expenditures on a monthly basis.

Despite the safeguards, we must consider the possibility that people neglected to milder illnesses for which there are no need to use medicine. The findings may indicate that poorer individuals tend to report illnesses less reliably. This is unlikely to be objectively true. Most surveys in developing countries that use externally observed measures of morbidity find higher levels of sickness among the poor. It could be suggested that the poor are particularly likely to modify their perception of illness in order to avoid economic costs, such as those of healthcare, associated with illness [20,21].

With respect to the pattern of drugs or service use, our findings illustrate that private health providers and self-treatment are commonly used while public providers are less so. This observation supports the results of previous studies [22,23]. Such a pattern is quite different from that found in the years prior to 1989, during which the health sector was subsidized entirely by the state and all people were treated free of charge using public facilities [5].

Based on these findings we can make the following observations. First, with the legalization of private practices, there has been an increase in the number of private healthcare providers and also in the availability of non-prescription drugs at markets or through pharmacies. Second, the decline in government resources for healthcare has led to a decrease in both the availability of subsidized drugs from public providers, and service quality at the low levels such as district health centre (DHC) and community health station (CHS) [23]. Finally, the elimination of free services has affected people's decisions regarding using of drugs and services.

The existence of a private healthcare sector has provided the wealthy with greater access to more and better health services and the poor with better access to medicines via self-medication or service through private pharmacies. However, the increase of self-medication or use of private services also reflects a reduction in the number of people contacting public services. Problems related to unsafe, improper or irresponsible use of drugs have also become more common as is described in a study from Tbilisi, Georgia that Self-treatment is cheaper than visiting a health care provider which explains the preference for self-treatment among the poorest quintile. Furthermore, weak enforcement of pharmaceutical regulations enables people to purchase even prescription drugs directly from pharmacies without a prescription. Thus, on the one hand, the high cost of medical care, the possibility of securing prescription drugs directly from the pharmacy, explain the popularity of the choice to self-treat. [20].

There are differences between the richest and poorest quintiles with respect to their use of medical provider. The poorest usually contact lower public providers like community health station (CHS) only when they get sick, whilst the richest typically contact higher level public providers, such as district health central (DHC) or province/centre hospital (P/CH). With respect to professional services, both poor and non-poor often used private services. Moreover, the number of episodes of self-treatment or no care was higher in the poorest group than in the richest group (see Table 1 and 2). These situations are also consistent with other studies. People in rural areas often choose self-medical or use private services before public services; and the poor use less public care and care at lower levels than the rich [22,24]. Such observations could relate to reduced access to State aid (in the form of partial subsidiary by the State to public healthcare centers) amongst the poor compared to the rich. Patients who use these facilities are typically wealthier and benefit from State-subsidized fees.

Among other factors that we found that might influence a person's choice of healthcare services was that as education level increased, the number of people choosing providers for healthcare also increased. This is similar to findings from other surveys [20,25], and implies that education has an influence on whether a population is strong and healthy based on their selection and investment in long-term routine healthcare. Education also appears to be a significant factor affecting choices for healthcare management. Women tend to use drugs or services more often than men. We recognize, however, that women and men have different healthcare problems and different perceptions of the importance of selecting and utilizing healthcare services [20]. The decision to use drugs or a medical provider is significantly influenced by the seriousness of the illness and by the SES of the household. A larger share of the patients who visit public healthcare providers and higher-level services (P/CHs) belong to the richest quintile or suffer from a serious illness (confined to bed). These analyses helped to realize that if a visit to the local healthcare facility does not help or if the person is diagnosed with a serious illness, they should be encouraged to contact a higher-level public hospital where more extensive diagnoses and appropriate treatment and care are available under doctors' supervision. Our research suggests that public providers still play the decisive role of the healthcare system in Vietnam.

Our findings also show that the cost of basic healthcare is of critical importance in the decision of when and what services to use. Income for the poor frequently derives from physical labor. When sick or confined to hospital due to illness, the poor are unable to work to earn money. Compounding the problem is the typical lack of savings by the poor. As a result, they will generally choose to ignore their illness or self-treat. When state of health has deteriorated to such an extent that they are incapacitated, they may fall even deeper into poverty as a result of the cost of healthcare and the corresponding loss of income whilst sick [26].

Our results show that the expenditure for a single illness episode treated in public facilities is greater than those in private facilities or by self-treatment (see Table 5). Our data also shows that expenditure for self-treatment is only 13% of total curative expenditure, while self-treatment episodes stand for 23% of total episodes where drugs and services were used. These shares are 27% and 10% respectively for public care; and 13% and 2% respectively for higher facility levels (P/CH) (Table 1 and 4). Those results support the discussion above that for more serious illnesses, people often chose public or higher public providers; for minor illnesses, people often undergo self-treatment or seek treatment from private providers. However, the evidence shows that the number of poor who utilize self-treatment (as well as their expenditure on such treatment) is higher than that of the non-poor, whereas use of public healthcare is less. In general, healthcare fees are the same for both the poor and non-poor, which mean that public sources mainly subsidize the rich rather than the poor.

**Table 5:** Average household expenditure per episode by providers and expenditure quintiles in Vietnamese dong

	Equalized household expenditure quintiles					Total
		
	Poorest	2	3	4	Richest	
Self treatment	11,854	15,595	18,495	29,945	32,063	19,616
providers (privatre+public)	31,755	42,504	46,344	40,127	63,059	45,887
Private providers	24,915	30,004	34,496	34,645	47,882	35,206
Traditional healer	39,290	49,659	52,881	38,421	26,224	38,236
Privatehealth care	23,645	28,730	32,432	33,995	52,379	34,803
Public providers	56,653	104,548	128,806	60,526	127,787	95,795
Commune health station	32,719	43,950	82,418	36,314	38,808	48,096
District health center	44,940	148,116	187,857	48,333	56,813	80,031
Province/central hospital	158,375	163,206	169,700	110,402	363,858	209,144
Total	23,428	33,997	38,228	38,354	58,179	39,191

Table 5 shows that the average household expenditure per episode of illness is less for self-treatment (919,616 VND) and for private providers (35,206 VND) than for public providers (95,795 VND). The average household health expenditure for a single illness episode is higher for hospital treatment than for district health centres and commune health stations.

HH is the abbreviation of household; p-value for the comparison  between the lowest and highest quintile groups; *** denotes significant at the 1% level; **denotes  significant at 5% level. Percentage shares are shown within brackets.

Taking into account the limitations of the study, we consider the distance to healthcare providers, either in physical units or in time, has generally been found to be associated with utilization of health services. Unfortunately, our data are likely to underestimate this association and are unable to paint a full picture of all factors that might influence a person's choice of healthcare services.

A major concern in is the substantial difference in access to different healthcare providers between the rich and poor. Part of this difference can be attributed to the transition from a socialist system to a market economy. However, during the time of our study the government has made considerable progress in developing and supporting programs for providing healthcare for the poor. In particular, the Government issued a program providing for and supporting the socio-economic development of the extreme rural areas of the country [27]. A major components of the government's program were the introduction of healthcare insurance and the public funding of healthcare expenses targeted specifically at the poor [28,29]. This progress could be expected to affect our findings. One of the objectives of the Government's program is to reduce the burden healthcare among the poor households in communes with special difficulties and to decrease the gap between poor and rich.

The results of this study should be of interest to policy-makers and healthcare professionals who are formulating healthcare policies. Of particular importance are the methods to reduce self-treatment and no-treatment. Our research has also identified several other significant issues. These include the management of private practices and maintaining public healthcare providers at all levels, particularly at the basic levels (district, commune) where the poor seek care more than the rich. Healthcare at the basic level is also vital because it is mostly utilized by the poor who find it very difficult and costly to access health facilities at higher levels. If we can improve the quality of such services, then it can help improve the treatment quality for a large portion of people, including the seriously ill in both the poor and non poor groups. The findings of this study could also provide a background for further studies and strategic policy-making on healthcare utilizations and healthcare financing.

## Competing interests

We, the authors, declare that there are no financial or non-financial conflicts of interest (political, personal, religious, academic, ideological, intellectual, commercial or any other).

## Authors' contributions

We are co-authors in this paper. Each author has participated sufficiently in the study to take responsibility for appropriate portions of the content as follow: NTBT: The first author, who designed the questionnaire, was responsible for monitoring the interview process, performing the statistical analysis, drafting and revising the manuscript CL: The second author, who participated in the planning of the study, was involved in drafting and revising the manuscript. LL and NTKC: The third and fourth authors, who participated in the planning of the study, and in the revisions of the manuscript. All co-authors read and approved the final manuscript.

## Pre-publication history

The pre-publication history for this paper can be accessed here:


